# Prevalence and risk factors for child labour and violence against children in Egypt using Bayesian geospatial modelling with multiple imputation

**DOI:** 10.1371/journal.pone.0212715

**Published:** 2019-05-30

**Authors:** Khaled Khatab, Maruf A. Raheem, Benn Sartorius, Mubarak Ismail

**Affiliations:** 1 Faculty of Health and Wellbeing, Sheffield Hallam University, Sheffield, United Kingdom; 2 Department of Engineering and Mathematics, Sheffield Hallam University, Sheffield, United Kingdom; 3 Department of Disease Control, Faculty of Infectious and Tropical Diseases, London School of Hygiene and Tropical Medicine, London, United Kingdom; Harvard TH Chan School of Public Health, UNITED STATES

## Abstract

**Background:**

The incidence of child labour, especially across developing nations, is of global concern. The use of children in employment in developing economies constitutes a major threat to the societies, and concerted efforts are made by the relevant stakeholders towards addressing some of the factors and issues responsible. Significant risk factors include socio-demographic and economic factors such as poverty, neglect, lack of adequate care, exposure of children to various grades of violence, parental education status, gender, place of residence, household size, residence type or size, wealth index, parental survivorship and household size. Egypt is the largest country in Africa by population. Although UNCIF 2017 reported that the worst forms of child labour in Egypt are concentrated in domestic work, forced begging and commercial sexual exploitation, the situation has received little attention. There are still very few studies initiated specifically to look at child labour in domestic service in Egypt and those that exist have been limited in the scope of their methodology. Geographical coverage and research for child labour in Egypt is also limited, as are accurate statistics and data. There was, therefore, a strong case for looking again at the domestic child labour phenomenon in Egypt, especially after the Demographic Health Survey (DHS) released the first data about child labour in Egypt in 2014. This study builds on the few findings of earlier work, and broadens coverage by including advanced methods and geographical effects of this problem.

**Objectives:**

This study focuses on identifying socio-demographic, economic and geospatial factors associated with child labour participation.

**Methods:**

We used the 2014 Egypt Demographic and Health Survey (EDHS) from the Ministry of Health and Population in Egypt, with the record of 20,560 never-married children aged 5–17 years engaging in economic activities, in and out of their home. The data focused on demographic and socio-economic characteristics of household members. Multivariate Bayesian geo-additive models were employed to examine the demographical and socio-economic factors for children working less than 16 hrs; between 16 and less 45 hrs; and over 45 hrs weekly.

**Results:**

The results showed that at least 31.6% of the children in the age group from 5–10 were working, 68.5% of children aged 11–17 years were engaged in child labour for a wage, and 44.7% of the children in the age group from 5–10 were engaged in hazardous work. From the multivariate Bayesian geo-additive models, female children (with male children as reference category) working at least 16 hrs (OR: 1.3; with 95% CI: 1.2–1.5) were more likely to be engaged in child labour than girls working 16 to 45 hrs (OR: 1; 95% CI: 0.3–1.5). Children born to women without formal education, in non-hazardous jobs, irrespective of the hours spent at work, were more likely to be involved in child labour (52.9%, 56.8%, 62.4%) compared to children of mothers with some level of education. Finally, children who have experienced psychological aggression and physical punishment are more likely to be used as child labour than those without such experience across the job types and hours spent. North-eastern Egypt has a higher likelihood of child labour than most other regions, while children who live in the Delta are more engaged in hazardous work.

**Conclusion:**

This study revealed a significant influence of socio-demographic and economic factors on child labour and violence against children in Egypt. Poverty, neglect, lack of adequate care and exposure of children to various grades of violence are major drivers of child labour across the country. The spatial effect suggests the need to give more attention to some areas that have high rates of child labour, such as the Nile Delta, Upper Egypt, and North-eastern Egypt.

## Introduction

In 1989, the General Assembly of the United Nations issued Resolution 44/25, the Convention on the Rights of the Child, which defines a child as a human being below 18 years of age. This convention emphasised the need to seek to protect children from performing any work that could be hazardous, interfere with their education, or damage their health or physical, mental, spiritual, moral or social development [1]. It also required Member States to take legislative, administrative, social and educational measures to ensure such protection. In addition, these states are obligated, according to this convention, to set a minimum age for employment, determine an adequate system of working hours and working conditions and impose appropriate penalties to ensure the effective application of these conditions.

The International Labour Organisation (ILO) reported in 2004 that in 1999–2000 overall poverty in Egypt rose to 20.2%; with the poverty rate highest in urban Upper Egypt (36.33%), followed by rural Upper Egypt (34.68%), but lowest in the Metropolitan region (9.01%). Khatab (2012) reported that at least 12 million people could not satisfy their basic food and non-food needs [2, 3].

Statistics (2010) indicate that there are around 11 million children under 15 in Egypt, and around 1.6 million work in child labour (ages 6 to 15) [4]. In 2015, the World Food Program and the European Union reported that the number of employed minors in Egypt had jumped to at least 2.7 million. Most of them work six days a week, for an average of 12 hours a day. They constitute an additional source of income for their families, who depend on that income to provide for one-third of their expenditures. Approximately 78% of working children are employed in the countryside, and most of them are female. Between 1 and 1.5 million are employed in agricultural labour. Of all working children, 84% live in rural areas and 16% in urban areas. Some recent statistics indicate that children who were engaged in child labour have experienced punishment and abuse, largely physical or verbal [4, 5].

Egypt has agreed to the ratification of the International Labour Treaty No. 138 of 1973 in addition to many international treaties which eliminate the criminal economic exploitation of children and aim in the long term for the total elimination of child labour. Egypt has also signed International Labour Treaty No. 182 of 1999, which is complimentary to Treaty No. 138, which urges the elimination of the worst forms of child labour initially, with the goal of eliminating all child labour. All forms of child labour are included in this agreement which also emphasises the importance of free basic education for children [4]. According to the UN, the child has the right to be protected from economic exploitation and from performing any work that is likely to be hazardous or to interfere with the child's education or be harmful to the child’s health or physical, mental, spiritual, moral or social development [6]. The legal employment age in Egypt is 18, the age when children should finish secondary school, while 21–22 is the age when they should finish their first degree at university.

Child labour is a narrower concept than children in employment. It is defined by the ILO Minimum Age Convention, 1973 (No. 138) and the ILO Worst Forms of Child Labour Convention, 1999 (No. 182) [4]. The definition used here is also based on Ministerial Decree 118 of 2003 issued by the Minister of Manpower and Migration, the national legislation that defines the occupations that children under the age of 18 are not allowed to engage in [7]. Despite this, the distribution of the population by age is such that a relatively high percentage of the population is young: those below the age of 15 represent about 37.5% of the total population, and of this 37.5%, 7% of Egyptian children below the age of 15 were engaged in child labour in 2009 [8]. Egypt is the fourth highest country for child labour, according to the World Bank, 2015 [5].

The major contributing factors to child labour in Egypt are poverty, deteriorating economic conditions and increasing inflation, which means that the situation is likely to worsen [8]. The poverty index measures severe health deprivation by the ratio of individuals who are not estimated to live to age 40. The 2004 Human Development Report (HDR) states that 3.1% (2.2 million people) of the total population of Egypt lives on less than USD 1 per day [9].

**Fig 1:**
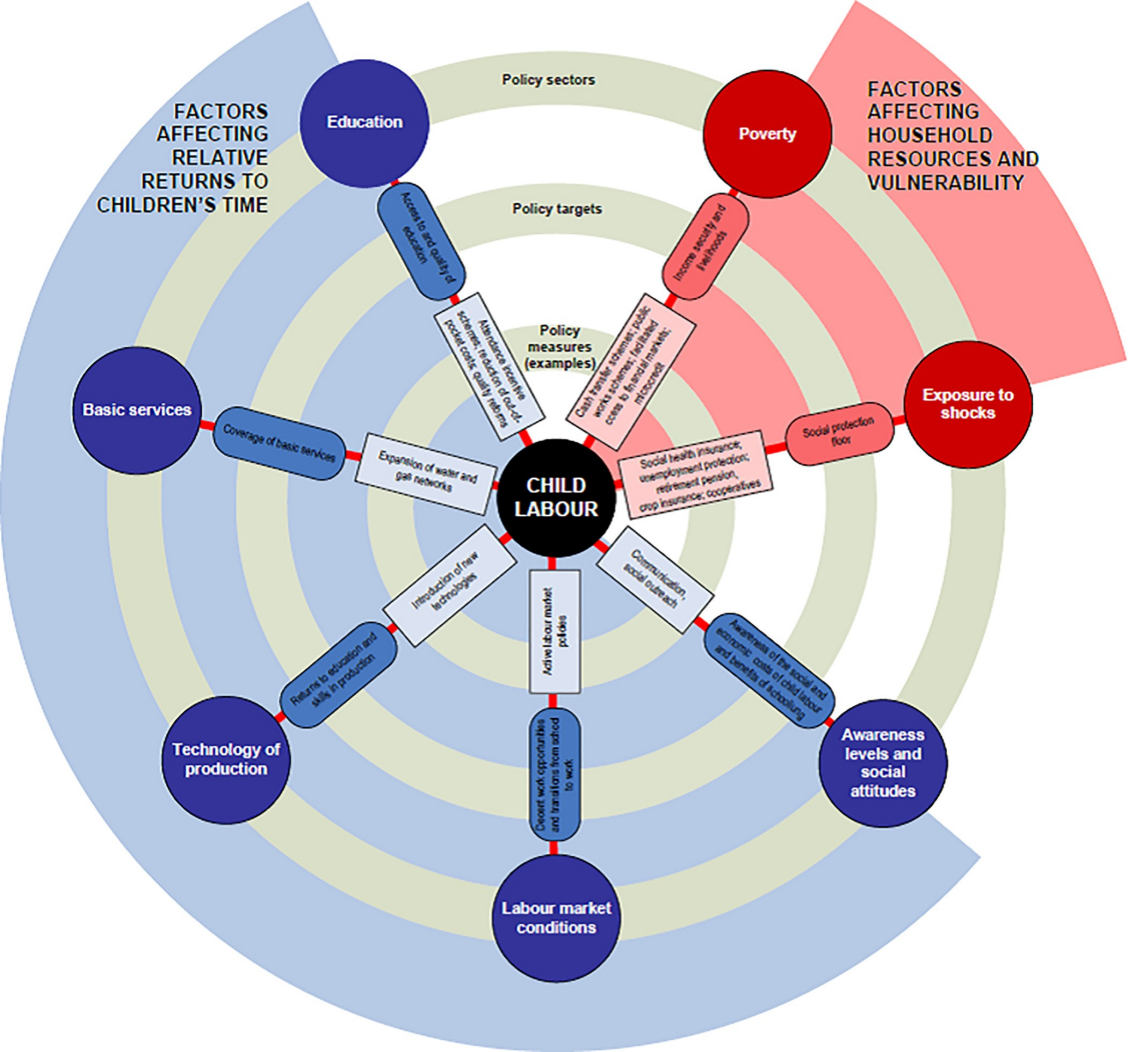
Factors influencing household concerning child labour (Source: Understanding child's work, a joint LIO-UNICEF-the world bank report, 2017).

Fig 1 shows the risk factors influencing households concerning child labour and factors affecting relative returns to children’s time. It shows that child labour is a symptom of poverty, but other factors also affect child labour [10]. These include work and schooling and how children or the household allocate time between schooling and work to increase household income.

The Central Agency for Public Mobilisation and Statistics (CAPMAS) reported that there are 12 million Egyptians who are homeless, of whom 1.5 million are living in cemeteries [2, 3]. It has been reported also that the number of people living below the poverty line in Egypt increased to 26.3% of the population in 2013 compared to 16.2% in 2000. Most of this increase has affected rural areas [11, 12]. The report showed that the urban frontier governorates had the lowest poverty rate with 11.4%, while rural Upper Egypt governorates showed the highest poverty rate with 49.4%. Upper Egypt is home to 25% of the country’s population, and at least 66% of people living there are extremely poor. It was found that, 37% of illiterate people were poor while only 8% of those who finished university were poor. Among large households with more than 10 members, 67% are poor[11].

This outlines the main challenges that parents and their children are facing, which allows us to understand the link between poverty, population density, size of household, restrictions in access to goods and services and lack of education, and how all of these factors have contributed to the increase of child labour in this region.

It is difficult to find accurate statistics on child workers or accurate studies that have discussed this problem properly. Therefore, there is an urgent need for a study that can describe this problem accurately, discuss the causes of it and provide some suggestions that may help decision-makers to handle this issue. Due to the negative effect of this phenomenon on the society, it is important to precisely investigate this problem further and thoroughly, using the results to improve outcomes and the situations for children [13]. This study, therefore, focuses on identifying socio-demographic, economic and geospatial factors associated with child labour participation.

## Materials and methods

The analysis in this work is based on data obtained from the 2014 Egypt Demographic and Health Survey (EDHS) **(S1 Data)** (Supporting Information) which is the most recent data on child labour in Egypt. The Survey was conducted by Egypt’s Ministry of Health and Population and the National Population Council in collaboration with Macro International [8]. As this was a secondary data analysis of open access data, ethics approval was not required. The sample for the 2014 EDHS was designed to provide estimates of population and health indicators, welfare indicators, and other indicator rates for the country as a whole and for six major subdivisions (Urban Governorates, urban Lower Egypt, rural Lower Egypt, urban Upper Egypt, rural Upper Egypt, and the Frontier Governorates) [8]. The sample likewise allows for estimates of most key indicators at the governorate level.

To allow for separate estimates for the major geographic subdivisions and the governorates, the number of households selected from each of the major sectors and each governorate was disproportionate to the size of the population in the units [8]. Thus, the dataset was weighted before proceeding with the analysis.

### Study area and data

The EDHS allows for an assessment of several key aspects of the welfare of Egypt’s children. Questions were included on birth registration and living arrangements and the survival status of parents. Data was also collected on the prevalence of injuries and accidents and disabilities among young children. A child’s access to education is critical, and the EDHS obtained information on both the level of pre-school education among young children and children’s participation in primary and secondary school.

The survey also looked at the extent of child labour and at the practices used in disciplining children. In the 2014 EDHS, the first step in the administration of the child labour and child discipline modules involved the identification of a single child age 1–17 years for whom questions in the modules would be asked depending on the child’s age. If the household included more than one child in the age range, the child for whom the modules were administered was selected using a Kish grid. The name of Leslie Kish, the Hungarian born American statistician who was one of the world’s leading experts on survey sampling. If the selected child was 5–17 years, the child discipline module was administered for the child. To account for the selection of one child per household, the child discipline data was weighted. The weight is based on the *de jure* population of children age 1–17 years [8].

### Description of outcome variables

The EDHS considered never-married children aged 5–17 years involved in economic activities inside or outside the home according to the child’s age and a number of hours worked. The MICS programme has defined thresholds based on the child’s age and the number of hours a child worked during the week to classify children’s involvement in economic activities [14]. The MICS programme was developed in the 1990s by the United Nations Children’s Fund (UNICEF) in collaboration with the World Health Organization (WHO), UNESCO, the United Nations Statistics Division, the United States Agency for International Development (USAID), the London School of Hygiene and Tropical Medicine, and the United States Centers for Disease Control and Prevention (CDC). This study used the ILO classifications which are based on the number of hours worked per week, in three groups: A) ≤16 hrs a week; B) >16<45 hrs a week; and C) ≥45 hrs a week.

The economic activities were classified into three groups: A) non-hazardous wage work; B) hazardous wage work; and C) household work. Work was considered hazardous if it involved carrying heavy loads, working with dangerous tools or operating heavy equipment, working at heights, working with chemicals or explosives, exposure to dust, fumes, gas, extreme heat or humidity, loud noise or vibrations, or any other working conditions considered to be bad for the child’s health and safety (EDHS, 2014)

### Risk factors and covariates

**Fig 2:**
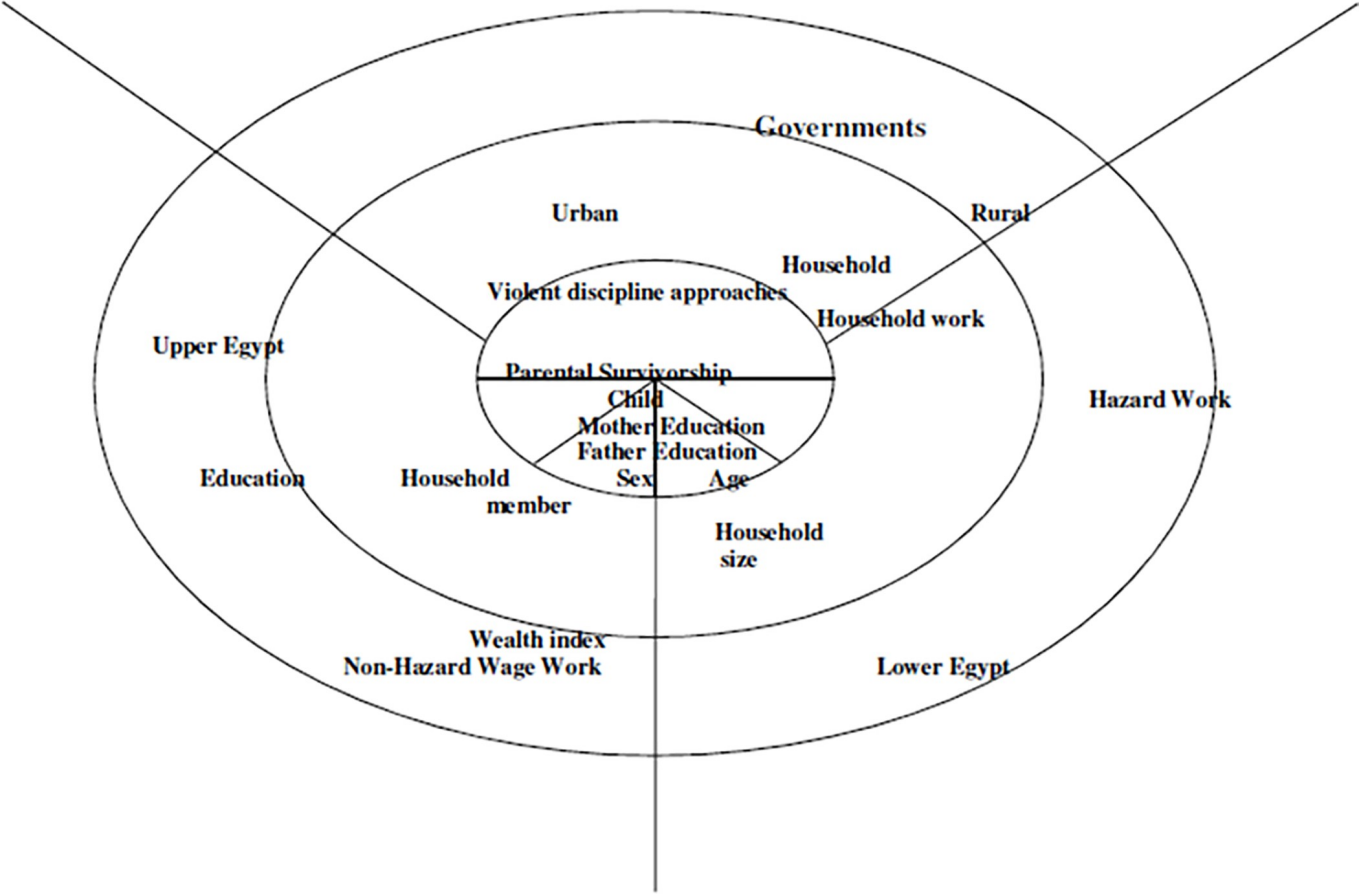
A multilevel risk factors of child labour that applied in this study.

We considered the following socio-demographic factors and the associated risk factors of child labour as explanatory variables: child’s age (5–17 years), sex, household size, place of residence, wealth index, mother’s education, father’s education, parental survivorship and violent discipline approaches against children. The wealth index was used as proxy for the socio-economic position of the household because EDHS did not collect information on household income and expenditure. Egypt comprises 27 governorates, which were categorised by EDHS into 7 areas: Urban governorates, Lower Egypt urban, Lower Egypt rural, Upper Egypt urban, Upper Egypt rural and Frontier governorates. However, in spatial analysis, we have used 27 governorates to investigate the spatial effects in the prevalence of overlap economic activities of children at the state level. This was achieved using a geo-additive semi-parametric multinomial model [15]. Fig 2 shows the multilevel risk factors of child labour that applied to this study and how these factors affected child labour on different levels.

### Statistical methods

Let *Y*_*ijk*_ and *π*_*ijk*_ represent the type of work and probability of working hours respectively. Let working hours of at least 15 hrs (*k* = 1); working hours falling between 16 and 45 hrs a week be (*k* = 2), and the working hours of over 45 hrs a week be (*k* = 3).

$$Y_{ij}∼MN(P_{ij,}N_{ij})$$ (1)

We assume that *Y*_*ijk*_ is distributed as a multinomial distribution, such that: *Y*_*ij*_~*MN*(1,*π*_*ijk*_) where *π*_*ijk*_ = (*π*_*ij*1_,*π*_*ij*2_,*π*_*ij*3_)′. Given some categorical covariates, *Z*_*ij*_, metrical covariates, *υ*_*ik*_ and state-specific random effect, *S*_*ik*_, the probability can be modelled thus:

Pijk=ηijkSkηijk=exp(ηijk)∑1kexp(ηijk);∀k=1,2,3 (2)

The predictor, *η*_*ijk*_ is given by *η*_*ijk*_ = *z*_*ij*_*β*_*k*_+*f*_*k*_(*υ*_*ij*_)+*S*_*ik*_ where *η*_*ijk*_ is a known response function with a logit link function, *β*_*k*_ which is the vector of the regression parameters (explanatory variables such as gender, place of residence, etc.) and *f*_*k*_ is a smooth function for the metrical covariates (child’s age) which were assumed to be nonlinear in some previous studies for each of the status categories, k [16–18]. We have included these variables as nonlinear metrical covariates in the early stage of this study; however, the pattern did not show exactly the significance level of each category. Therefore, we used these covariates as linear effects instead to assess the significance level of each category. We set the last category (over 45 hrs) as reference category.

The random effects, *S*_*ik*_ are district or sub-district specific factors, which can be split into spatially structured variation (*θ*_*ik*_) and unstructured multinomial heterogeneity (*ϕ*_*ik*_), such that, *S*_*ik*_ = *θ*_*ik*_+*ϕ*_*ik*_. P-spline priors were assigned to the functions; *f*_1_,*f*_2_,…*f*_*p*_ whereas, a Markov random field prior was used for f(s_i_) [19, 20].

To estimate model parameters, we applied a fully integrated Bayesian approach. Though the estimation method with this model is difficult, the estimated posterior odds ratios (OR) that were produced could be understood as similar to those of normal logistic models. The analysis was carried out using version 2.1 of the BayesX software package, which certifies Bayesian inference based on Markov chain Monte Carlo (MCMC) simulation technique [19, 21–23].

Both the descriptive statistics and chi-square tests were carried out to examine the level of associations between predictors, confounders, and outcome variables using version 14 of STATA, and p-values of less than 0.05 were considered statistically significant. The multinomial logistic regression model was used to determine the degree of associations between the outcome variables (a type of work indicators) and all the predictors. Posterior Odds Ratios (OR) and their 95% confidence intervals (CI) were estimated.

#### Multiple imputations analyses (missing data)

This dataset has a significant proportion of missing data although the missingness is concentrated only in a few variables. At this stage, we will assess the monotone patterns and the joint probability of missing across variables; thereafter we will identify the potential predictors of each variable that requires modelling [24–27]. The patterns of missingness were obtained to investigate the monotone patterns of missing and to assess the appropriate modelling that can handle these missing values.

One analytic option is to use only the dataset with complete observations, or we can replace the missing values through a process termed ‘imputation’. The simplest imputation replaces the missing value with mean or median value for that variable; however, this is not a desirable process, especially when one is examining the relationships between variables.

However, the more sophisticated method, termed ‘multiple imputations’, predicts missing values for a variable using existing values from other variables. The predicted values, called ‘imputes’, are substituted for the missing values, resulting in a full data set called an ‘imputed data set’ [27, 28]. This method imputes dataset using standard procedures for complete data and combines the results from these analyses.

No matter which complete data analysis is used, the process of combining results from different data sets is essentially the same. We use the data from units where both (*Y*,*X*) are observed to learn about the relationship between Y and X, then use this relationship to complete the data set by drawing the missing observations from, *X*|*Y*. This process is completed at least N = 5 times, giving rise to N complete data sets. Then each of these imputed data sets is analysed and we combine the results using specific rules. It does not attempt to estimate each missing value through simulated values but rather to represent a random sample of the missing values [29, 30]. Multiple imputations inference involves three distinct phases as follows:

1. Create imputed data sets which are plausible representations of the data.
2. Perform the chosen statistical analysis on each of these imputed data sets by using standard procedures.
3. The results from the complete data sets are combined ‘average’ for the inference to produce one set of results.

Analyses based on multiple imputed data will avoid bias only if enough variables that predict the missing values are included in the imputation models. Thus, including as many predictors as possible tends to make the missing-at-random assumption more plausible [31]. However, including more than 25 predictors will increase the variance explained in the prediction equations [32].

Multiple imputations are a more appropriate choice as a solution to missing data problems as this method represents a good balance between quality of results and ease of use. It has been shown to perform favourably compared to other methods in a variety of missing data situations [33]. It can also produce unbiased parameter estimates which reflect the uncertainty associated with estimating missing data, and has been shown to be robust to departures from normality assumptions and to provide adequate results in the presence of low sample size or high rate of missing data.

The results of some previous studies using the most commonly used multiple imputation methods, Expectation Maximization (EM-algorithm)[34] and the Monte Carlo Markov chain (MCMC) method, showed that there was no significant difference between EM algorithm and the MCMC method for item imputation, and that the number of items used for imputation has little impact.

However, in this study, we applied the MCMC method based on pseudo-random draws and this allowed us to obtain several imputed data. MCMC can be used with both arbitrary and monotone patterns of missing data. It is known as a collection of techniques for simulating random draws from difficult probability distributions via Markov chains. MCMC is also especially useful in Bayesian statistical analyses and for solving difficult missing-data problems [28, 35, 36].

#### Assumptions about missing data

If EDHS data contained observations that were missing completely at random, the observations would constitute a random sample of the complete dataset. Multiple imputations assume that the observed variables are predictive of the missing values and that the data is missing at random. Missing data is said to be missing at random (MAR) if the probability that it is missing does not depend on unobserved data but may depend on observed data. MCAR can be viewed as a particular case of MAR.

If the subjects are withdrawn from the study for ethical reasons, missing would not be MAR. This type of missing-data mechanism is called missing not at random (MNAR). For such missing data, the reasons for its missingness must be accounted for in the model to obtain valid results. We looked at missing data patterns and also assessed the extent of missing in the variables that were included in the analysis. Nonlinear relationships were treated using semiparametric models (e.g. generalised additive models (GAMs)) [22]. It was important to include the outcome variable (in this case, economic activities) as a predictor in the imputation model because failing to do so will dilute the associations between the outcome and the other variables[37]

## Results

### Prevalence and sectoral distribution of child labour

We used the 2014 Demographic and Health Survey (EDHS) from the Ministry of Health and Population in Egypt, with the record of 20,560 children aged 5–17 years engaging in economic activities, in and out of their home. The data focused on demographic and socio-economic characteristics of household members

**Table 1** shows statistics on child work and education in Egypt according to the UNESCO Institute for Statistics, 2015. While **Table 2** shows an overview of children’s work by sector and activity in Egypt according to the US Department of Labour report, 2016 [38]

**Table 1:** Statistics on child work and education in Egypt according to the UNESCO Institute for Statistics, 2015.

Children	Age	Per cent
Working (%) Population	5-14yrs	6.7(993,417)
Attending school (%)	6–14	88.1
Combining Work and school (%)	7–14	6.3
Primary Completion Rate (%)		10.8

**Table 2:** An overview of children’s work by sector and activity in Egypt (Bureau of labour Statistics, 2016).

Sector	% of Children work
Agriculture: Production of cotton Caring for livestock Fishing activities	52.2%
Industry: Quarrying limestone Making bricks Working in carpentry Construction Working in aluminium factories	16.5%
Services: Domestic Work Repainting automobiles street work including selling goods, collecting garbage, and sweeping	30.4%

**Table 1** shows that at least 6.7% of the children in the age group from 5–14 are working, 88.1% of children aged 6–14 years are attending school, and 6.3% engage in child labour while attending school. Only 10.8% complete primary school. **Table 2** provides an overview of children’s work by sector and activity in Egypt. It shows that over 52% of children are working in agriculture followed by 30.4% who are engaged in service sectors [38, 39].

**Table 3** shows the distributions of the missing values among the risk factors.

**Table 3:** Distributions of the missing values among the risk factors.

Risk factors	Valid cases	Missing values
Sex of child	35756	84520(70%)
Age of eligible children under 20years	35756	84520(70%)
Residence	120276	0
Household size	120276	0
Wealth index	120276	0
Mother’s Education	31450	88826(73.9%)
Father Education	120263	13(0.001%)
Parental Survivorship	120276	0
Phsholoical punishments	79927	40349(33.5%)
Physical punishments	79923	40390(33.6%)
Severe Physical Punishments	79886	40390(33.6%)

### Distribution of factors analysed in child labour in Egypt (DHS 2014)

**Table 4** presents the distribution of socio-demographic factors relating to child labour including violence against children aged 5–17 years in Egypt with respect to the weekly length of working hours in jobs that are hazardous, non-hazardous and household-based.

**Table 4:** Distribution of factors analysed in child labour in Egypt (DHS 2014).

	Non-Hazard Wage Work (% and N)			P-value	Hazardous Wage Work (% and N)			p-value	Household Work (% and N)			p-value
Sex of child	<16hours	16-less 45hours	45hours and above	0.002	<16hours	16-less 45hours	45 hours and above	0.001	<16hours	16-less 45hours	45 hours and above	0.001
Male	735(64.4)	479(69.6)	265(73.8)		169(56.3)	8(61.5)	13(43.3)		7455(51.2)	451(38.9)	69(46.6)	
Female	406(35.6)	209(30.4)	94(26.2)		131(43.7)	5(38.5)	17(56.7)		7103 (48.8)	708(61.1)	79(53.4)	
**Age of children**												
5-10years	360(31.6)	154(22.4)	68(18.9)	**0.001**	134(44.7)	4(30.8)	12(40)	**0.001**	5719(39.3)	308(26.6)	51(34.5)	**0.001**
11–15	439(38.5)	273(39.7)	141(39.3)		114(38)	6(46.2)	10(33.3)		5514(37.9)	500(43.2)	55(37.2)	
over 15	342(30)	260(37.8)	150(41.8)		52(17.3)	3(23.1)	8(26.7)		3325(22.8)	350(30.2)	42(28.4)	
**Residence**												
Urban	223(8.7)	253(16.6)	188(24.7)	**0.001**	53(8.2)	8(32)	8(14.5)	**0.001**	10930(33.7)	666(26.3)	69(20.8)	**0.001**
Rural	2341 (91.3)	1270(83.4)	574(75.3)		590(91.8)	17(68)	47(85.5)		21468(66.3)	18171(73.7)	262(97.2)	
**Household size**												
Small household	1025(40)	716(47)	343(45)	**0.001**	303(47.1)	16(66.7)	28(50)	**0.2**	16672 (51.5)	1185 (46.7)	148(44.7)	**0.001**
Medium household	1374(53.6)	770(50.5)	387(50.7)		319(49.6)	8(33.3)	28(50)		14868(45.9)	1252(49.3)	178(53.8)	
Large household	165(6.4)	38(2.5)	33(4.3)		21(3.3)	0	0		857(2.6)	101(4)	5 (1.5)	
**Place of Residence**												
Urban governorates	38(1.5)	85(5.6)	76(10)	**0.001**	4(0.6)	0(0)	0(0)	**0.001**	3663(11.3)	206(8.1)	13(3.9)	**0.001**
Lower Egypt	1310(51.1)	694(45.6)	309(40.6)		413(64.2)	16(66.7)	55(100)		15951(49.2)	802(31.6)	151(45.8)	
Upper Egypt	114(47.3)	735(48.3)	374(49.1)		219(34.1)	8(33.3)	0(0)		12560(38.8)	1493(58.8)	166(50.3)	
Frontier Governorates	2(0.1)	9(0.6)	3(0.4)		7(1.1)	0(0)	0(0)		224(0.7)	37(1.5)	0	
**Wealth index**											0.003	
Poorest	1754 (68.4)	819(53.8)	339(44.4)	**0.001**	275 (42.8)	8(32)	2(3.6)	**0.001**	7597(23.4)	830(32.7)	76(23)	**0.001**
Porrer	479(18.7)	347(22.8)	196(25.7)		143(22.2)	0(0)	13(23.6)		7328(22.6)	658(25.9)	109(32.9)	
Middle	196(7.6)	152(10)	89(11.7)		167(26)	9(36)	32(58.2)		6136(18.9)	364(14.3)	73(22.1)	
Richer	96(3.7)	111(7.3)	75(9.8)		52(8.1)	0(0)	8(14.5)		5596(17.3)	413(16.3)	31(9.4)	
Richest	38(1.5)	94(6.2)	64(8.4)		6(0.9)	8(32)	0(0)		5742(17.7)	273(10.8)	42(12.7)	
**Mother’s Education**												
No	582(52.7)	374(56.8)	201(62.4)	**0.001**	111(38.5)	3(23.1)	1(3.2)	**0.001**	4443 (31.7)	508(46.6)	39(30.2)	**0.001**
Primary	148(13.4)	124(18.8)	67(20.8)		58(20.1)	5(38.5)	6(19.4)		1937(13.8)	184(16.9)	20(15.5)	
Secondary	361(32.7)	149(22.6)	50(15.1)		100(34.7)	5(38.5)	24(77.4)		6374(45.5)	368(33.8)	70(54.3)	
Higher	14(1.3)	12(1.8)	4(1.2)		19(6.6)	0(0)	0(0)		1246(8.9)	30(2.8)	0(0)	
**Father Education**												
No	754 (29.4)	441(29)	203(28.7)	**0.08**	188(29.3)	4(16.7)	9(16.1)	**0.03**	7840(24.2)	678(26.7)	70(21.1)	**0.001**
primary	673(26.3)	411(27)	207(29.2)		223(34.7)	8(33.3)	16(28.6)		8741(27)	676(26.6)	90(27.2)	
Secondary	1043(40.7)	602(39.6)	282(39.8)		198(30.8)	12(50)	27(48.2)		13234(40.9)	1056(41.6)	155(46.8)	
Higher	93(3.6)	68(4.5)	16(2.3)		33(5.1)	0(0)	4(7.1)		2576(8)	128(5)	16(4.8)	
**Parental Survivorship**												
Both Alive	1130(44.1)	637(41.8)	315(41.3)	**0.3**	327 (50.9)	15(60)	30(53.6)	**0.67**	14952 (46.2)	1132(44.6)	145(43.8)	**0.001**
Father deceased	52(2)	6(0.4)	6(0.8)		13(2)	0(0)	0(0)		116(0.4)	22(0.9)	7(2.1)	
Mother deceased	14(0.5)	32(2.1)	25(3.3)		0(0)	0(0)	0(0)		626(1.9)	66(2.6)	4(1.2)	
Both deceased	0(0)	0(0)	0(0)		0(0)	0(0)	0(0)		13(0)	0(0)	0(0)	
don’t know/missing	1368(53.4)	849(55.7)	416(54.6)		303(47.1)	10(40)	26(46.4)		16691(51.5)	1318(51.9)	175(52.9)	
**Violent discipline approaches**												
**1)Psychological aggression**												
Yes	1402(90.2)	578(92.3)	204(89.1)	**0.002**	380(77.1)	17(100)	11(52.4)	**0.36**	22552(93.8)	1137(90.6)	148(98)	**0.001**
No	153(9.8)	48(7.7)	25(10.9)		113(22.9)	0(0)	10(47.6)		1478(6.2)	118(9.4)	3(2)	
												
**2)Physical punishment**												
Yes	961(61.8)	356(56.9)	133(58.1)	**0.08**	380(77.1)	17(100)	11(52.4)	**0.002**	15433(64.2)	690(55)	60(40)	**0.001**
No	594(38.2)	270(43.1)	96(41.9)		113(22.9)	0(0)	10(47.6)		8597(35.8)	564(45)	90(60)	
**3) Severe physical punishment**												
Yes	927(59.6)	376(60.1)	138(60.3)	**0.9**	395(80.1)	8(47.1)	20(95.2)	**0.001**	15366(64)	677(53.9)	79(52.7)	**0.001**
No	628(40.4)	250(39.9)	91(39.7)		98(19.9)	9(52.9)	1(4.8)		8634(36)	578(46.1)	71(47.3)	

The following factors were significantly associated with non-hazardous wage work (see **Table 4)**: gender of a child (P = 0.002); residence type, household size, place of residence and wealth indices (P = 0.001 each); violent discipline approach, especially: psychological aggression (P = 0.002) and physical punishment (P = 0.08). However, the following factors were not significant in this category: parental education, parental survival and severe punishment. For the hazardous wage work, the non-significant factors were household size, parental survivorship, and psychological aggression against children. While, gender, the age of children, place of residence, wealth indicators, mother’s education, physical and severe punishments were significant (each with P = 0.001). Father’s education had a slight effect. For household work, all the factors were highly significant (each with P = 0.001).

The percentage of male children involved in child labour is higher than that of female children, irrespective of the job type (non-hazardous, hazardous and household-based work) and the length of job, except that female children in a household job who are working at least 16 hours weekly have a higher percentage (61.1%) than male children (53.4%). Also, children from rural communities are more likely to be used as child labour than their peers from urban cities, with higher percentages across the board of job type and hours spent at work.

Children living in a medium-sized household and involved in non-hazardous jobs apparently have the highest working rates (53.6%, 50.5% and 50.7%) compared to their peers in both small and large households, irrespective of the hours spent at work.

Children from Lower and Upper Egypt are engaged in non-hazardous and household jobs and they have the highest percentage across the three periods of a job. However, for hazardous jobs, children from the Lower Egypt area seem to have the highest percentage of exposure (64.7%, 66.7%, 100%).

For the wealth indicators, children from the poorest homes are more likely to be in non-hazardous jobs, with the following percentages (68.4%, 53.8% and 44.4%) respectively compared to their peers from wealthier homes. However, for the hazardous work category, there are relatively few participants working 16–45 hrs and also relatively few working >45 hrs. The poorest children, who work at least 45 hrs in both hazardous and household jobs, have a higher percentage of working as child labour than their peers.

Children born to women without formal education in non-hazardous jobs, irrespective of the hours spent at work, are mostly working as child labour with the following percentages: 52.7%, 56.8% 62.4%, compared to children of mothers with some level of education. Children whose parents are both alive seem to be more likely to work as child labour in household job conditions than their peers without living parents.

Finally, children who have experienced psychological aggression and physical punishment are more frequently working as child labour than those without such experience, across the job types and hours spent. The same with those who have experienced severe physical punishment compared to their counterparts who have not had such experiences.

#### Associated risk factors

**Table 5** displays the multinomial regression results for socio-demographic factors relating to child labour among children aged 5–17 years in Egypt with respect to the weekly length of working hours in jobs that are hazardous, non-hazardous and household-based. The table presents the estimated effects of the categorical variables: sex of child, residence's type, household size, place of residence, wealth index, parental education, parental survivorship and violent discipline approaches.

**Table 5:** Associate risk factors with child labour in Egypt (DHS 2014).

	Non Hazard Wage Work		Hazardous Wage Work		Household Work	
Sex of child	<16hours	16-less 45hours	<16hours	16-less 45hours	<16hours	16-less 45hours
Male	1	1	1	1	1	1
Female	1.3(1.2–1.5)	1(0.9–1.2)	0.6(0.3–1.5)	1.6(1.4–2.7)	1.2(0.7–2)	1.7(0.9–3.2)
**Age of eligible children under 20years**						
5-10years	1	1	1	1	1	1
11–15	0.5(0.4–0.8)	0.8(0.5–1.2)	0.7(0.3–2.2)	1.6(0.3–8.9)	1.6(0.7–3.7)	2.3(0.9–5.6)
over 15	0.4(0.3–0.6)	0.7(0.5–1.035)	1.36(0.3–3.2)	1.3(0.8–1.1)	0.7(0.5–0.8)	0.35(-0.6–0.8)
**Residence**						
Urban	1	1	1	1	1	1
Rural	0.15(0.11–0.2)	0.3(0.2–0.4)	0.8(0.2–2.9)	0.3(0.05–2.03)	0.95(0.4–2.6)	1.7(0.6–5.2)
**Household Size**						
Small household	1	1	1	1	1	1
Medium household	0.86(0.7–0.9)	1.4(1.3–1.6)	1.12(0.5–2.8)	0.8(0.18–3.4)	0.9(0.5–1.6)	0.8(0.6–1)
Large household	0.01(0–0.03)	0.08(0–0.09)	1.6(0.5–2)	1.2(0–2.2)	0.96(0.5–1.8)	1.4(0.7–2.6)
**Place of Residence**						
Urban governorates	1	1	1	1	1	1
Lower Egypt	0.53(0.09–3)	0.18 (0.01–2)	0.7(0.1–2)	0.2 (0–1.2)	2.5(0.9–6.8)	1.2(0.4–3.5)
Upper Egypt	0.6(0.1–3.2)	0.3(0.02–3)	0.8(0.3–1.8)	0.3(0.2–3.4)	2.3(0.8–6.5)	2.5(0.8–7.4)
Frontier Governorates	0.02(0.002–0.23)	0.16(0.01–2)	0.1(0–1.3)	0.4(0.01–2.3)	3.2(0.6–16)	4.9(0.9–26)
**Wealth index**						
Poorest	1	1	1	1	1	1
Porrer	0.6(0.5–0.7)	0.8(0.4–1.6)	1.93(0.29–12.8)	0.2(0.1–2)	1.2(0.53–2.8)	0.9(0.4–2)
Middle	0.16(0.13–0.2)	0.3(0.13–0.9)	2.3(0.4–10.07)	0.3(0.8–1.2)	1.4(0.6–3.4)	0.8(0.3–2.1)
Richer	0.02(0.014.0.03)	0.24(0.09–0.6)	0.58(0.5–1.3)	0.15(0–1.3)	4(1.2–15)	3.4(0.8–13
Richest	0.014(0.09–0.02)	1(0.26–3.8)	1.46(1.2–6.2)	0.7(0.2–1.8)	2.6(0.7–9.5)	2(0.5–8)
**Mother’s Education**						
No education or complete primary	1	1	1	1	1	1
Complete secondar or Higher	4.5(2.3–8.5)	2.2(1.2–4.5)	0.4(0.1–1.2)	0.01(0–0.9)	0.8(0.4–1.5)	0.7(0.4–1.6)
**Father Education**						
No education or complete primary	1	1	1	1	1	1
Complete secondar or Higher	1.87 (1–3.4)	1.6(0.8–3)	0.4(0.1–1.5)	0.02(0–1.1)	0.8(0.3–1.9)	0.8(0.3–5.6)
**Parental Survivorship**						
Both Alive	1	1	1	1	1	1
Father deceased	1.02(0.3–3.5)	0.54(0.5–2.3)	0	0	1.5(0.6–4.2)	2.1(0.8–5.8)
Mother deceased	0.5(0.2–1.2)	0.4(0.13–1.2)	1.64(0.2–22)	1.02(0–1.4)	0.2(0.08–0.5)	0.6(0.3–1.5)
**Violent discipline approaches**						
**1)Psychological aggression**						
Yes	1.53(0.7–3.3)	3.7(1.5–9.5)	0.012(0–0.4)	0.01(0–0.2)	1.3(0.6–3.3)	1.08(0.4–2.8)
No	1	1	1	1	1	1
						
** 2)Physical punishment**						
Yes	0.8(0.4–1.3)	0.6(0.3–1.06)	6.4(1.9–21)	1.43(0–1.5)	9.3(4.2–20.8)	7.9(3.4–17)
No	1	1	1	1	1	1
** 3) Severe physical punishment**						
Yes	0.8(0.4–1.3)	1.02(0.5–1.9)	0.4(0.1–1.3)	0.001(0–0.02)	0.9(0.5–1.7)	0.5(0.3–1)
No	1	1	1	1	1	1

Columns 1 & 2 present the odds of children working ‘less than 16 hours’ weekly as against those working ‘between 16 and 45 hours’ weekly, under non-hazardous working conditions. For example, the results show that female children (with male children as reference category) working less than 16 hrs (OR: 1.3; with 95% CI: 1.2–1.5) are less likely to be working as child labour than those working 16 to 45 hrs (OR: 1.6; 95% CI: 1.4–2.7). Also, for those in household jobs, female children working between 16 and 45 hrs (with OR: 1.7; 95% CI: 0.9–3.2) are more likely to be working as child labour than those working at less than 16 hrs weekly (OR: 1.2; 95% CI: 0.7–2). Finally, under the hazardous working condition, female children working 16–45 hrs (OR: 1.6 and 95% CI of 1.4–2.7) are more at risk than those working less than 16 hrs weekly (OR: 0.6; 95% CI: 0.3–1.5).

Similarly, children aged 11–15 (OR: 0.5 and 95% CI: 0.4–0.8) are more likely to be at risk than those aged over 15 years (OR: 0.4; 95% CI: 0.3–0.6) under the non-hazardous working condition for less than 16 hrs and for 16–45 hrs, although, the OR does not seem significant in either age group. For children in a household job, 11–15 year-olds are more likely to be at risk of being used as child labour (OR: 2.3 and 95% CI: 0.9–5.6) compared to those who are over 15 and are in 16–45 hrs weekly job category. For hazardous jobs, children aged over 15 years who are working less than 16 hrs (OR: 1.36; 95% CI: 0.3–3.2) are more at risk of working as child labour compared to their counterparts who are under the age of 15.

Rural children under 16–45 hrs are more likely to be at risk of being used as child labour than the children working less than 16 hrs a week in non-hazardous jobs. Urban children in non-hazardous and hazardous jobs are more likely to be at risk of being used as child labour compared to rural children, regardless of the number of hours. Rural children under household jobs are more likely to be affected by child labour compared to urban children.

Children living in medium-sized households who are working 16–45 hrs weekly (with OR: 1.4; 95% CI: 1.3–1.6) have an increased likelihood of being used as child labour in non-hazardous jobs than in hazardous and household jobs. However, children in large households, and working less than 16 hrs a week under hazardous conditions (OR: 1.6; 95% CI: 0.5–2) are likely to have a higher risk compared to others in the two other working environments.

The poorer children working under the hazardous condition for less than 16 hrs weekly (OR: 1.93; 95%CI: 0.29–12.8) are likely to be most at risk compared to their colleagues subjected to the two other working conditions. The same experience goes for middle-class children (OR: 2.3; 95%CI: 0.4–10.07). However, for the children from the richer and the richest families, those in household jobs and working less than 16 hrs weekly, or 16–45 hrs are more likely to be at risk of child labour than other children who are in the other two working conditions.

Half-orphans whose fathers are dead tend to be more often working as child labour in non-hazardous and household-based jobs than those whose mothers are deceased, irrespective of the hours of labour. However, for hazardous jobs, children with deceased mothers are more likely to be at risk than those with deceased fathers.

Children who are exposed to psychological aggression and are working 16–45 hrs weekly (OR: 3.7; 95%CI: 1.5–9.5) in a non-hazardous job are more likely to be at risk of being used as child labour than their counterparts subjected to the two other working conditions. The same with children subjected to severe physical punishment. However, the risk likelihood is highest for children in household jobs who are subjected to physical punishments, especially those working less than 16 hrs weekly (OR: 9.3; 95%CI: 4.2–20.8).

#### Spatial effects

**Fig 3:**
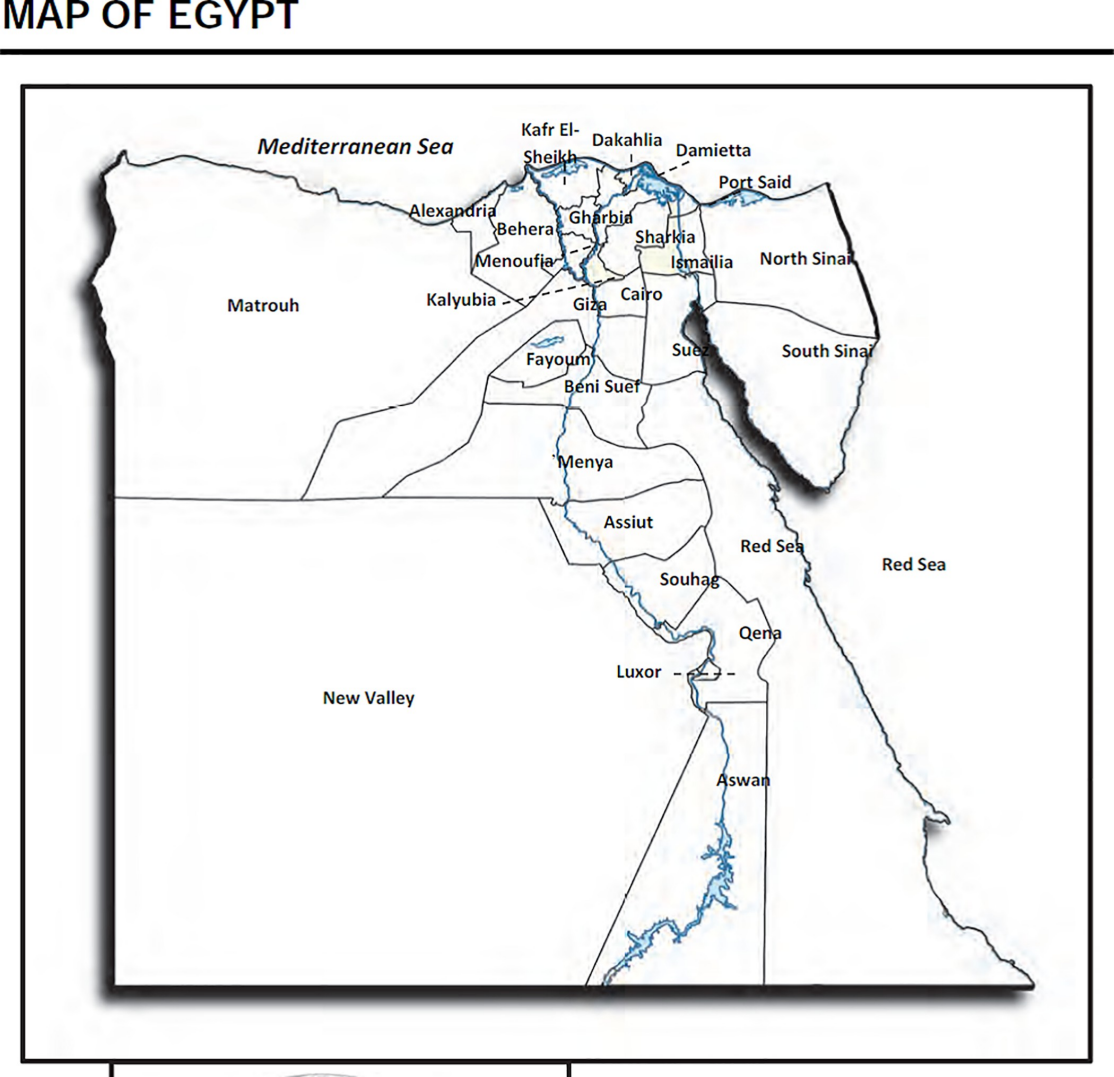
Map of Egypt.

**Fig 4:**
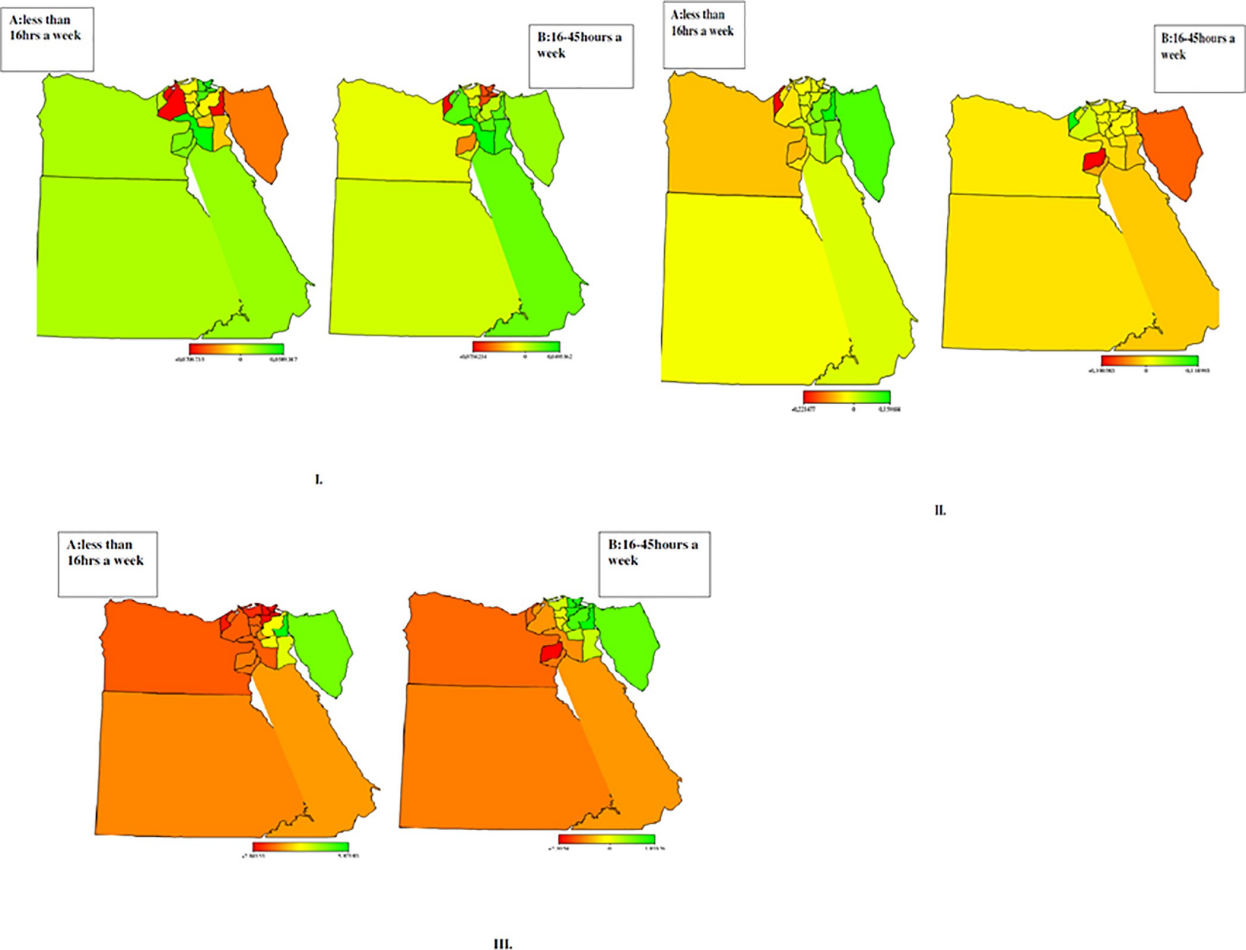
Maps of Egypt showing the spatial effects (posterior OR) on child labour: Fig 4I. (A) less than 16 hrs a week Vs. (B) 16–45 hrs a week engagement in Household work, Fig 4II. (A) less than 16 hrs a week vs. (B) 16–45 hrs a week engagement in wage work, Fig 4III. (A) less than 16 hrs a week Vs. (B)16-45 hrs a week engagement in Hazard work. The red area indicates a negligible effect for this child labour within these areas, while the green area reflects a strong effect of a child involved in different types of work in these regions and the yellow area indicates that there are almost no cases found in these regions.

Fig 3 shows the map of Egypt (EDHS 2014). Fig 4 shows the structured spatial effects of child labour. The results confirmed evidence of regional differences in the likelihood of a child being involved in different types of work. From the graph, it is clear the north-eastern region and upper (rural-urban) of Egypt have a higher likelihood of child labour than most other regions. Using the Frontier governorate as a reference, children residing in rural Upper Egypt have the highest risk of working in all three employment types. Hazardous work is likely in Delta, Canal, Ismailia and other neighbouring cities; whilst lower and upper Egypt are more affected by household work. Similarly, the likelihood of being required to do non-hazardous work is higher in lower and upper Egypt.

## Discussion

This study emphasises the significance of seeking child protection against engaging in any job or task that could be hazardous, interfere with education or damage the health or physical, mental, spiritual, moral or social development of a child, in line with the UN convention. We reviewed how poverty, declining economic conditions and rising inflation continue to worsen the situation across various parts in Egypt.

We cross-classified the economic activities of the children aged 5–17, who are never-married and are of school age, based on the number of hours worked per week, subject to ILO classifications against the socio-demographic and spatial factors to determine the level of association between these factors and the nature of jobs these children are working in. We went further to determine which of the socio-economic and spatial factors and the associated risk factors are likely to expose these children to the risks inherent in the various kinds of work they are engaged in.

From the results, we found that gender, age, household size, place of residence, region, violent discipline method, parental education and survivorships and wealth status are all predisposing factors for child labour in different working conditions.

We found that the percentage of male children who were in non-hazardous jobs worked longer hours than female children. However, more than 61% of female children engaged in household jobs working between 16 and 45 hrs a week compared to their male counterparts (39%). Likewise, female children spend more hours weekly (over 45 hrs) in household jobs compared to male children. Another study that used the National Child Labour Survey (2010) found that about 30% of employed children are working in hazardous industries (7.7%), such as mining and construction, or hazardous occupations (1.7%), or for long hours (20%) [7].

The percentage of children living in a medium-sized household and working over 16 hrs a week is higher compared to those both in the small and large households. Children living in Upper and Lower Egypt, those from the poorest homes, those born to women without formal education and those who have experienced both psychological and physical punishments have a higher percentage in all categories.

We found that children aged 11–15 are more at risk of being used as child labour than those aged over 15 years. Children between 11–15 are more at risk because they are more vulnerable, especially when they are not living with their parents, as is the case with most of these children in this dataset. Therefore, they are most likely to be sent to work to support themselves and their relatives, compared to their counterparts who are over 15, who are more mature and therefore less vulnerable to being used as child labour without their consent. However, children in the age group of 11–15 are more engaged in housework and non-hazardous work, compared to their counterparts who are over 15. The same goes for rural children, who have higher chances of working than their urban colleagues. This is consistent with previous studies which used DHS data and found that children in rural areas are much more likely to work and less likely to be in school [40]. Children from poorer homes, those whose fathers are dead and those subjected to psychological aggression and physical punishment are more likely to be sent into child labour. A previous study has found that the effect of household wealth on child labour is statistically significant, but the significant effects are almost always negative, as expected [40]. The main finding was how household wealth influences the decision between work and school for children.

The majority of children under the age of 17 and adults in rural areas of Upper-Egypt are living in great poverty. At least three-quarters of the 9.2 million children are living in great poverty in rural areas. However, recent years have seen a movement in poverty in urban cities, which replicates the ‘impact of the prolonged economic stagnation that started in 2011’ [7]. Regional inequalities continue to be part of the country’s landscape, with Upper Rural Egypt showing poverty rates three times as high as metropolitan Egypt [5]. The spatial effect suggests the need to contribute more to some areas that have high proportions of child labour, such as the Nile Delta, Upper Egypt, and North-eastern Egypt. The higher proportion of child labour in these areas compared with other areas is triggered by the high-density population and high rates of poverty [16].

From our findings, one could argue that there is considerable evidence of child labour in Egypt and that socio-demographic and spatial factors greatly predispose the majority of the children to engage in it.

### Conclusion

We have presented Bayesian geospatial modelling with multiple imputation models for child labour and violence against child issues in Egypt. These types of epidemiological studies are relatively rare. Most of the previous studies were limited to exploratory analysis of child health only [9, 16, 41].

We have established evidence of the presence of child labour and the impacts of socio-demographics and their associated risk factors for child labour across Egypt

This study is novel because the association between geospatial factors, socio-demographic factors and child labour has not been investigated before in Egypt. The findings reveal a significant influence of socio-demographic and economic factors on child labour and violence against children in Egypt. Significant is that poverty, neglect, lack of adequate care and exposure of children to various grades of violence are major drivers of child labour across the country. North-eastern Egypt has a higher likelihood of child labour than most other regions, while children who live in the Delta are more engaged in hazardous work. The government is therefore encouraged to work towards protecting the rights of children as enshrined in the United Nations conventions and to educate and empower children from less-privileged families.

### Strength and limitations of this study

As far as we know, this is the first time a study of this kind has been undertaken in Egypt on geospatial factors affecting child labour and the first which implements advanced modelling whilst imputing the missing values that usually affect the data of the child labour in developing countries. Using geospatial mapping and statistical imputation to handle large quantities of missing data is an important contribution to the field.

The study is conducted in line with the 2014 EDHS, which promotes the assessment of several key aspects of the welfare of Egypt’s children. However, the level of missing observations in the data is a major challenge in this study. Only a few studies have discussed child labour in Egypt due to the lack of the accurate data and statistics, and most of these did not use DHS data, neglected the spatial effects, and did not use the advanced statistical methods that we have used in this study. This is why it was difficult to compare our results with the previous studies. As the number of observations increases (20,560 observations were used), the power of the chi-square test increases. Obviously, as we have more data, we can be more certain about our effect size not being attributable to noise. Increasing the sample size tends to result in a smaller P-value only if the null hypothesis is false, which is not the case. For hazardous work, there are fewer observations, and that could also affect the analysis of this outcome variable. According to the EDHS, it was difficult to collect more data about such sensitive economic activities.

## Supporting information

S1 Data EDHS data 2014 on child labour. (SAV)
